# Detection of Intestinal Dysbiosis in Post-COVID-19 Patients One to Eight Months after Acute Disease Resolution

**DOI:** 10.3390/ijerph191610189

**Published:** 2022-08-17

**Authors:** Alexandre Soares Ferreira-Junior, Tais Fernanda Borgonovi, Larissa Vedovato Vilela De Salis, Aline Zazeri Leite, Amanda Soares Dantas, Guilherme Vedovato Vilela De Salis, Giuliano Netto Flores Cruz, Luiz Felipe Valter De Oliveira, Eleni Gomes, Ana Lúcia Barretto Penna, Gislane Lelis Vilela De Oliveira

**Affiliations:** 1 Microbiology Program, Institute of Biosciences, Humanities and Exact Sciences (IBILCE), Sao Jose do Rio Preto 15054-000, Brazil; 2 Food Engineering and Technology Department, Sao Paulo State University (UNESP), Sao Jose do Rio Preto 15054-000, Brazil; 3 Barretos Board of Health, Emergency Care Unit, Barretos 14780-900, Brazil; 4 BiomeHub Research and Development, Florianopolis 88054-700, Brazil

**Keywords:** post-COVID-19, intestinal microbiota, dysbiosis, antibiotics, sequelae

## Abstract

The intestinal microbiota plays an important role in the immune response against viral infections, modulating both innate and adaptive immune responses. The cytokine storm is associated with COVID-19 severity, and the patient’s immune status is influenced by the intestinal microbiota in a gut-lung bidirectional interaction. In this study, we evaluate the intestinal microbiota of Brazilian patients in different post-COVID-19 periods, and correlate this with clinical data and the antibiotic therapy used during the acute phase. DNA extracted from stool samples was sequenced and total anti-SARS-CoV-2 antibodies and C-reactive protein were quantified. Compared with controls, there were significant differences in the microbiota diversity in post-COVID-19 patients, suggesting an intestinal dysbiosis even several months after acute disease resolution. Additionally, we detected some genera possibly associated with the post-COVID-19 dysbiosis, including *Desulfovibrio*, *Haemophillus*, *Dialister*, and *Prevotella*, in addition to decreased beneficial microbes, associated with antibiotic-induced dysbiosis, such as *Bifidobacterium* and *Akkermansia*. Therefore, our hypothesis is that dysbiosis and the indiscriminate use of antibiotics during the pandemic may be associated with post-COVID-19 clinical manifestations. In our study, 39% (*n* = 58) of patients reported symptoms, including fatigue, dyspnea, myalgia, alopecia, anxiety, memory loss, and depression. These data suggest that microbiota modulation may represent a target for recovery from acute COVID-19 and a therapeutic approach for post-COVID-19 sequelae.

## 1. Introduction

Coronavirus Disease 2019 (COVID-19), an infectious disease caused by Severe Acute Respiratory Syndrome Coronavirus 2 (SARS-CoV-2), spread rapidly worldwide and was declared a Global Pandemic on 11 March 2020 [1,2]. According to the World Health Organization, SARS-CoV-2 has already infected more than 587 million people worldwide, with 6,428,661 deaths [3]. Brazil was one of the most affected countries by COVID-19, with 34,171,644 confirmed cases and 681,550 deaths [4].

The COVID-19 comprises a wide spectrum of clinical manifestations, ranging from asymptomatic to critically ill patients characterized by respiratory and/or multi-system organ failure [5,6,7]. Even though the respiratory tract is mainly affected, the gastrointestinal tract can also be affected, with nausea, vomiting, abdominal pain, and diarrhea [8]. In addition, several patients have been experiencing long-term sequelae and symptoms after the resolution of the acute phase, including myalgic encephalomyelitis and chronic fatigue syndrome [9]. The influence of the immune system on both acute and long COVID-19, has been demonstrated in several studies [6,8,9,10]. In the acute form, a massive cytokine production, also known as a cytokine storm, has been associated with the progression to severe cases, acute respiratory distress syndrome, multi-system organ failure, and coagulation dysfunctions [11]. The cytokine storm in COVID-19 is characterized by an overproduction of tumor necrosis factor-alpha (TNF), interleukin-1 (IL-1), IL-2, IL-6, and interferons [12,13]. In chronic fatigue syndrome, the pathophysiology seems to be related to immune dysfunctions as well, including changes in cytokine profile, immunoglobulin levels, and in T and B-cell phenotypes [14].

The intestinal microbiota plays an important role in the immune response against viral infections, modulating both innate and adaptive immune responses [15]. Growing evidence suggests that short chain fatty acids (SCFAs) produced by balanced microbiota are essential for the immune system’s development and maturation, besides being fundamental in combatting against respiratory infections [15,16]. Infections and medications, mainly antibiotics, promote modifications in the richness and diversity of the lung and intestinal microbiota, leading to dysbiosis [16,17]. The bidirectional interaction, also known as the gut–lung axis, has been associated with different outcomes in respiratory tract infections, including influenza and respiratory syncytial virus, emphasizing the importance of the communication between these mucosal compartments [16,18].

In COVID-19, increasing age and comorbidities are known risk factors for predicting the severe form of the disease, and they have been associated with intestinal dysbiosis, which might explain the development of such severe cases on those conditions [19,20,21,22]. The available data support that during the COVID-19 infection, the gut–lung axis, and its influence on the immune response, plays an important role in a progression to a cytokine storm, multi-system organ failure, and long-term COVID-19 syndromes [8,14,15]. To date, some studies have shown that intestinal dysbiosis, detected in acute COVID-19, was characterized by a predominance of opportunistic microorganisms and decreased beneficial commensals, even after the respiratory symptoms’ resolution [23,24,25]. In addition, recent studies suggest that dysbiosis could be involved in immune response magnitude and disease severity, as well as in persistent symptoms after COVID-19 resolution [26,27]. In this observational study, we evaluated the intestinal microbiota in Brazilian patients, in different post-COVID-19 periods, and correlated the results with the clinical data and antibiotic therapy used during the acute disease.

## 2. Materials and Methods

### 2.1. Post-COVID-19 Patients and Control Group

The Research Ethics Committee from the Institute of Biosciences, Humanities and Exact Sciences from Sao Paulo State University approved this study (Process number 4,310,336/2020). It was performed in accordance with the Declaration of Helsinki, and all participants signed the informed consent form.

One hundred and forty-nine post-COVID-19 patients, one to eight months after having acute COVID-19, were enrolled, from October to December 2020, for this study. They provided the previous positive tests for SARS-CoV-2, detected by qPCR after nasopharyngeal swab collection. Patients were enrolled at the Clinical Analysis Laboratory of the Institute of Hematology in Sao Jose do Rio Preto/Brazil, including 98 females and 51 males, aged 18 to 82 years old (42.5 ± 14.5 years). In addition, patients were classified as follows, according to the acute disease in: (1) Asymptomatic (*n* =10): Positive SARS-CoV-2 test and no symptoms; (2) Mild (*n* = 117): symptoms such as fever, cough, anosmia, ageusia, diarrhea, without dyspnea; (3) Moderate (*n* = 10): Clinical evidence of lower respiratory tract disease, non-invasive oxygen support (oxygen saturation < 94%); and Severe (*n* = 12): invasive oxygen support, admission to the intensive care unit, orotracheal intubation [28,29]. They were also classified according to patients without antibiotic (ATB) therapy (COVID-19 group: *n* = 35 patients) and with ATB therapy during the acute disease phase (COVID-19+ATB group: *n* = 114 patients).

Seventy-one subjects, 51 females and 20 males, aged 18 to 79 years old (46.1 ± 16.6 years), selected for other projects prior to the COVID-19 pandemic, were included in this study as the control group. Exclusion criteria for the control group included use of anti-inflammatories, immunosuppressant drugs, antibiotics, laxatives, probiotics and vaccination in the 30 days preceding the sample collection, as well as gastrointestinal surgeries, inflammatory bowel diseases, and chronic diarrhea. The stool and DNA samples from the post-COVID-19 and control subjects were stored at −80 °C until the analysis.

After the informed consent, 8 mL of peripheral blood was collected, and stool samples were requested within 3–5 days. Serum total anti-SARS-CoV-2 antibodies and C-reactive protein (CRP) concentrations were performed in post-COVID-19 patients by electrochemiluminescence and immunoturbidimetric assay, respectively, at the Clinical Analysis Laboratory of the Institute of Hematology.

### 2.2. DNA Extraction and Microbiota 16S Sequencing

DNA was obtained from 200 mg of stool samples from post-COVID-19 and control subjects by using QIAamp Fast DNA Stool Mini Kit (Qiagen, Hilden, Germany), according to the manufacturer’s instructions. For DNA libraries, two amplification steps (PCR) were used. The first PCR was performed using the specific primers 341F 5’-CCT ACG GGR SGC AGC AG-3’ and 806R 5’-GGA CTA CHV GGG TWT CTA AT-3’ [30]. The primers contained in their structure adapters based on those used for the TruSeq approach (Illumina Inc., San Diego, CA, USA). The second PCR inserted index sequences into the libraries, which enabled the identification of samples. The final PCR reaction was purified using a protocol based on magnetic beads (AMPureXP, Beckman Coulter, Pasadena, CA, USA), and the libraries were pooled for quantification. The quantification of libraries was performed by qPCR with the KAPA Library Quantification Kit for Illumina platforms (KAPA Biosystems, Wilmington, MA, USA). All 220 samples (149 patients/71 controls) were sequenced at the same time/sequencing run. The sequences generated by 16S sequencing were deposited at the National Center for Biotechnology Information (NCBI) repository (BioProject ID: PRJNA758913).

### 2.3. Statistical Analysis

All statistical analyses were performed using the R software package (v. 4.0.3) (R Core Team, Vienna, Austria, 2021). Microbiome data wrangling was carried out using the tidyverse (v. 1.3.0) and phyloseq (v. 1.34.0) R packages [31,32]. Libraries with less than 500 reads were removed. The beta diversity analysis included Principal Coordinate Analysis using Bray-Curtis dissimilarity and taxa proportions as input. PERMANOVA models were computed using the ATdonis function from the vegan (v. 2.5.7) R package [33]. The alpha diversity analysis included Shannon index assessment using non-parametric tests (Kruskal-Wallis or Wilcoxon rank-sum) as appropriate. Differential abundance analysis was performed using the corncob (v. 0.2.0) R package. Taxa present in less than 10% of samples were filtered out prior to this analysis. Confidence intervals for the taxa prevalence were computed using the exact method from the binom (v. 1.1.1) R package. The *p* values were adjusted for the control of false-discovery rate (FDR) at 5%, using the Benjamini–Hochberg step-up procedure [34].

## 3. Results

### 3.1. Characterization of the General Post-COVID-19 Population

First, we characterized our general post-COVID-19 population, without taking into account the disease severity classification during the acute phase. The collected post-COVID-19 time points ranged from one to eight months (four to 34 weeks) after the acute disease (~30 days post-COVID-19 = 38 patients; ~60 days post-COVID-19 = 30 patients; ~90 days post-COVID-19 = 18 patients; ~120 days post-COVID-19 = 50 patients; ~150 days post-COVID-19 = 10 patients; and ~240 days post-COVID-19 = 3 patients).

The body mass index (BMI) of the patients ranged from 17 to 43 kg/m^2^ (28.6 ± 5.4 kg/m^2^), with 3 patients underweight (BMI < 18.5), 38 within ideal weight (18.5–24.9 kg/m^2^), and 51 patients overweight (25–29.9 kg/m^2^), 57 obese (BMI > 30 kg/m^2^), among these 39 were obesity grade I (30–34.9 kg/m^2^), 14 grade II (35–39.9 kg/m^2^), and 4 had morbid obesity (BMI > 40 kg/m^2^). In the obese group, 8 patients had the severe and 7 had the moderate form of the disease. In addition to obesity, other comorbidities were reported for post-COVID-19 patients, including systemic arterial hypertension (21%), type 2 diabetes (7.4%), other heart diseases, such as arrhythmias, coronary artery disease and cardiac dispositive (5.4%), chronic lung diseases, including asthma (4.7%), chronic renal disease (2.7%), depression (3.3%) and autoimmune diseases (2%).

The serum quantification of total anti-SARS-CoV-2 antibodies ranged from 0.07 to 199.5 (69.5 ± 58.4), with 15 non-reactive patients (6 asymptomatic, 8 mild, 1 moderate). The CRP ranged from 0.1 to 6.3 mg/dL (mean ± SD = 0.59 ± 0.94 mg/dL) with 17 patients with results higher than 0.50 mg/dL, indicative of inflammatory/infectious process (1 severe, 2 moderate, 13 mild, 1 asymptomatic).

Table 1 summarizes the demographic, anthropometric, and clinical data from post-COVID-19 patients based on the disease severity classification during the acute phase, and demographic and anthropometric data from control subjects.

We also classified the general post-COVID-19 population in patients without ATB therapy (COVID-19 group: 23.5%) and patients with ATB therapy during the acute phase (COVID-19+ATB group: 76.5%). Thirty-five patients were included in the COVID-19 group, including 6 asymptomatic, 28 mild, and 1 moderate. One hundred and fourteen patients were included in the COVID-19+ATB group, among them 4 were asymptomatic, 89 mild, 9 moderate, and 12 severe.

Regarding clinical manifestations, Table 2 shows the distribution of the main symptoms reported by post-COVID-19 patients during the acute phase of the disease, the ATB therapy, as well as the percentages of patients with sequelae. Approximately 39% (N = 58) of patients reported clinical manifestations after resolution of the acute phase. The most common symptoms among the mild post-COVID-19 patients included fatigue, anosmia, anxiety, depression, myalgia, alopecia, memory loss, and depression, followed by muscle weakness, ageusia, tachycardia, sweating, parosmia, breathlessness, paresthesia, skin rashes, arthralgia, headaches, and dizziness. The rarest symptoms included muscle spasms, insomnia, nausea, polydipsia, inappetence, tremors, diarrhea, blurry vision, and changes in bowel functions. For moderate and severe patients, the most common post-COVID-19 sequelae were fatigue, sarcopenia, dyspnea, myalgia, cough, paresthesia, post-traumatic stress, memory loss, anosmia, ageusia, alopecia, and edema. The rarest sequelae included plegia, dysphonia, hypothyroidism, hyperinsulinemia, dyslipidemia, systemic arterial hypertension, and some patients still required oxygen support and pulmonary rehabilitation.

### 3.2. Detection of Intestinal Dysbiosis in Brazilian Post-COVID-19 Patients

**Figure 1:**
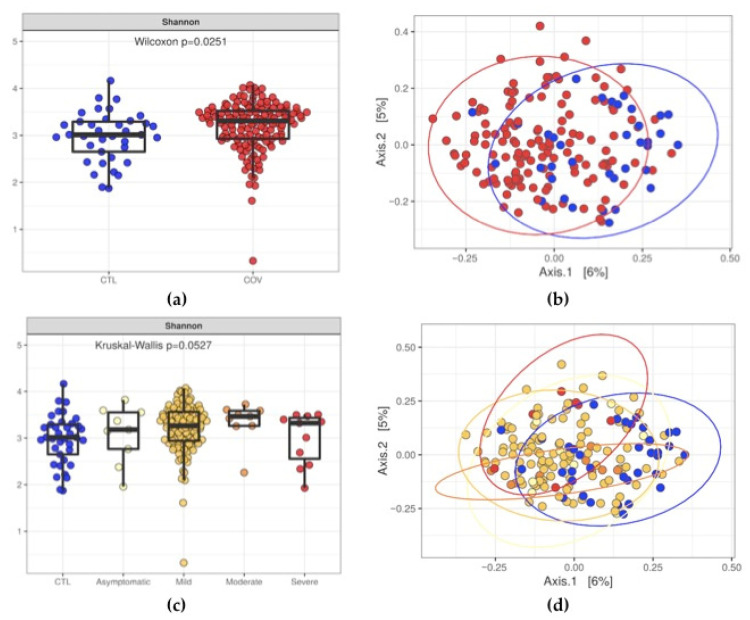
Diversity analysis of the intestinal microbiota from post-COVID-19 patients (COV) and control subjects (CTL). (**a**,**c**) Shannon indices showing the evenness of the intestinal microbiota, and (**b**,**d**) Principal Coordinate Analysis (PCoA) plots showing the differences in microbiota diversity between post-COVID-19 patients and control subjects.

**Figure 2:**
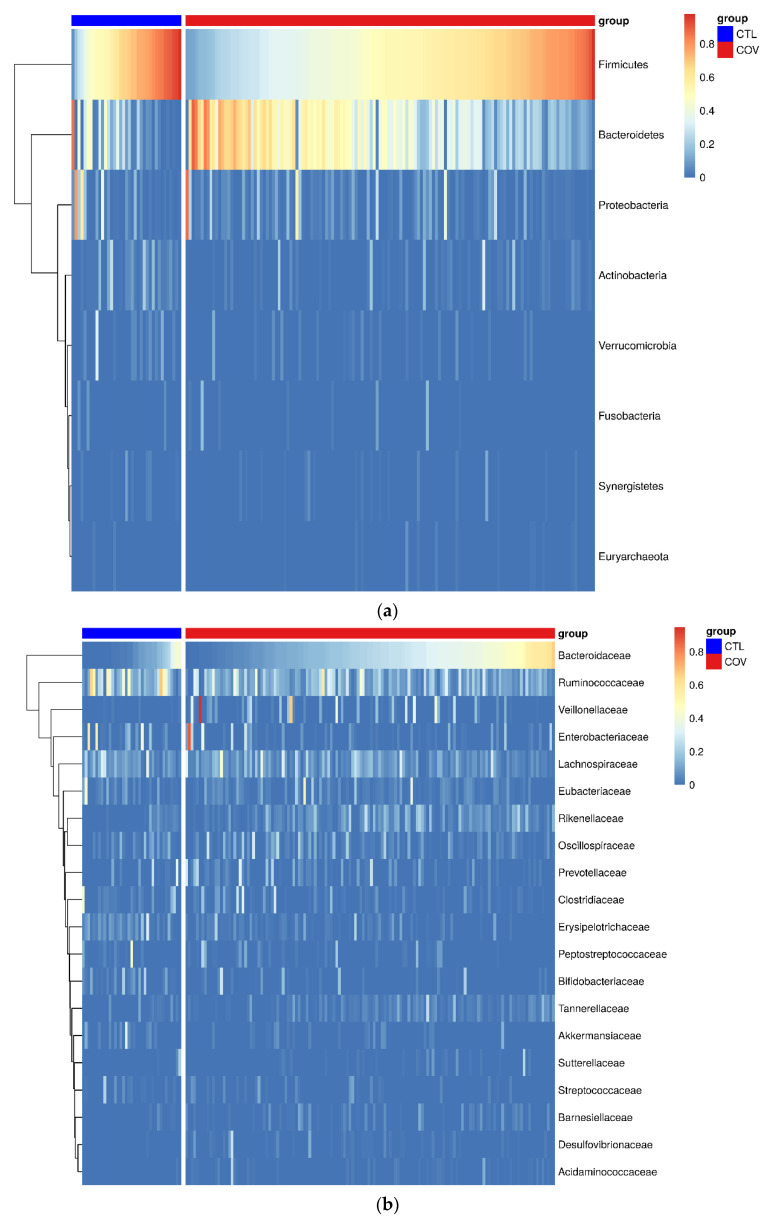
Compositional differences in intestinal microbiota in stool samples from post-COVID-19 patients (*n* = 149) and control subjects (*n* = 71). (**a**) Top eight phyla and (**b**) top 20 microbial families.

In order to evaluate the intestinal dysbiosis in patients after acute COVID-19, we sequenced the V3/V4 regions from bacterial 16S and performed the diversity analysis using the annotated operational taxonomic units (OTUs). According to the Shannon index, we observed differences (*p* = 0.0251) in evenness when we evaluated stool samples from post-COVID-19 patients and control subjects. Likewise, we also detected significant differences (*p* = 0.0015) in microbial diversity in post-COVID-19 patients (Figure 1a,b). When grouping patients into asymptomatic, mild, moderate, and severe, we did not observe differences (*p* = 0.0527) in microbiota evenness, However, we detected significant differences in microbiota diversity (*p* = 0.0015) using Bray-Curtis dissimilarity and taxa proportions to calculate beta diversity (Figure 1c,d). Figure 2 shows the heatmaps containing the top eight phyla (a) and top 20 microbial families (b) detected in stool samples from post-COVID-19 patients and control subjects.

Alpha and beta diversities were also calculated by allocating post-COVID-19 patients’ reads according to gender, age, and BMI; as a result, no significant differences were detected in any of these analyses. When grouping patients according to COVID-19 without ATB therapy and COVID-19+ATB during the acute disease, differences in beta diversity were not detected. Furthermore, differences in post-COVID-19 time points and correlations among microbiota, total anti-SARS-CoV-2 antibodies, and CRP were not observed.

### 3.3. Differential Genera Relative Abundance in Post-COVID-19 Patients

**Figure 3:**
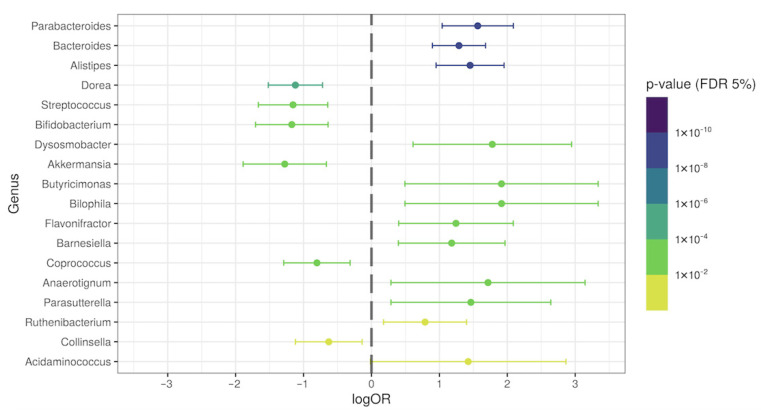
Differential genera relative abundance analysis of the intestinal microbiota in general post-COVID-19 patients (*n* = 149), compared with control subjects (*n* = 71).

With the purpose of investigating the presence of specific genera in our general population of post-COVID-19 patients, we evaluated the differential relative abundance in patients’ samples and compared it with the control group. The relative abundance percentages of the *Parabacteroides*, *Bacteroides*, *Alistipes*, *Dynosmobacter*, *Butyricimonas*, *Bilophila*, *Flavonifractor*, *Barnesiella*, *Anaerotignum*, *Parasutterella*, and *Acidaminococcus* genera were significantly increased (*p* < 0.05; logOR > 1) in the feces of post-COVID-19 patients, when compared with the control subjects. On the other hand, the *Dorea*, *Streptococcus*, *Bifidobacterium*, and *Akkermansia* genera were significantly reduced in post-COVID-19 (*p* < 0.05; logOR > −1) (Figure 3). Figure S1 shows the relative abundance of some overrepresented genera in post-COVID-19 patients. Figure S2 shows some underrepresented genera in post-COVID-19 patients.

### 3.4. Specific Intestinal Microbiota Signature in the COVID-19 Group without Antibiotic Therapy

**Figure 4:**
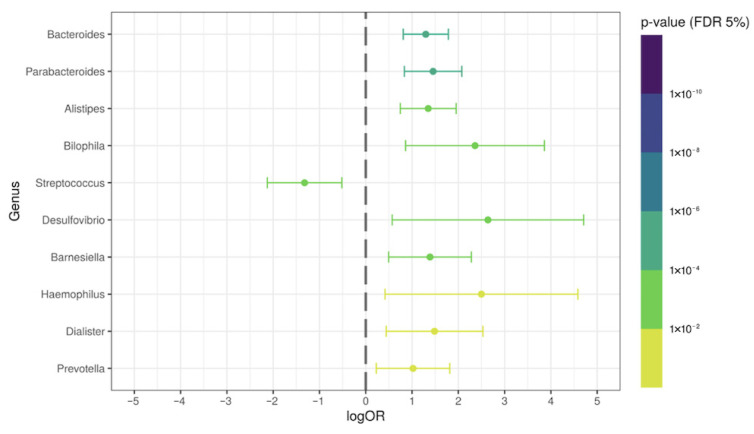
Differential genera relative abundance analysis of the intestinal microbiota in post-COVID-19 patients without ATB therapy (N = 35), compared with control subjects (N = 71).

To identify a possible specific signature of the intestinal microbiota after acute COVID-19 (disease-associated), we compared post-COVID-19 patients without ATB therapy with the control group. We observed some genera possibly associated with intestinal dysbiosis induced by the COVID-19. The relative abundance of the *Bacteroides*, *Parabacteroides*, *Alistipes*, *Bilophila*, *Desulfovibrio*, *Barnesiella*, *Haemophillus*, *Dialister*, and *Prevotella* genera were significantly increased (*p* < 0.05; logOR > 1) in the feces of those from the COVID-19 group. Interestingly, the *Streptococcus* genus was significantly decreased (*p* = 0.004; logOR = −1.32) (Figure 4). Figure S3 shows the relative abundance of specific overrepresented genera in post-COVID-19 patients without antibiotic therapy.

### 3.5. Antibiotic-Induced Alterations in Some Bacteria Genera in Post-COVID-19 Patients

**Figure 5:**
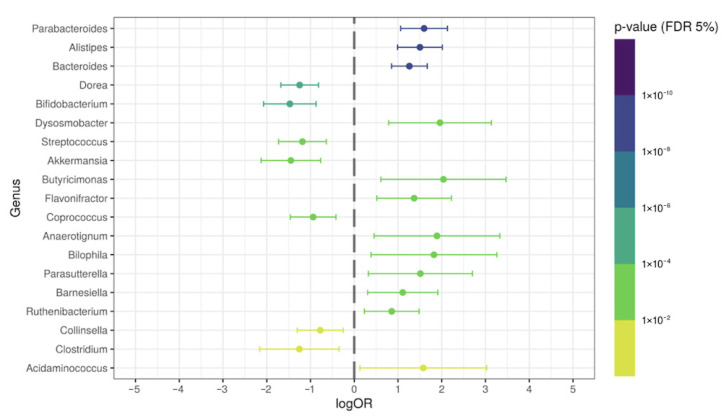
Differential genera relative abundance analysis of the intestinal microbiota in post-COVID-19 patients with ATB therapy (*n* = 114), compared with control subjects (*n* = 71).

**Figure 6:**
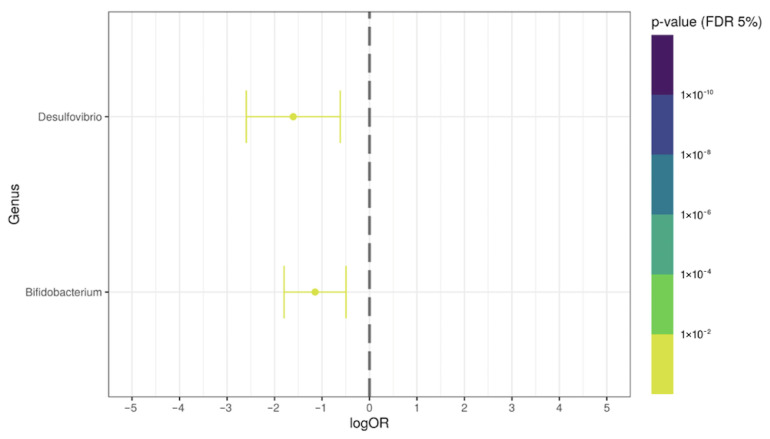
Differential genera relative abundance analysis of the intestinal microbiota in post-COVID-19+ATB group (N = 114), compared with post-COVID-19 group (N = 35).

Afterwards, in order to demonstrate the specific effect of the antibiotic therapy on the post-COVID-19 patients (antibiotic-associated), we evaluated the differential relative abundance between COVID-19+ATB versus control group, and COVID-19 group versus COVID-19+ATB. We observed some genera possibly associated with antibiotic-induced alterations in the microbiota from the post-COVID-19 patients. The relative abundance of the *Parabacteroides*, *Alistipes*, *Bacteroides*, *Dysosmobacter*, *Butyricimonas*, *Flavonifractor*, *Anaerotignum*, *Bilophila*, *Parasutterella*, *Barnesiella*, and *Acidaminococcus* genera were significantly increased (*p* < 0.05; logOR > 1) in the COVID-19+ATB group, when compared with the control subjects. In addition, the *Dorea*, *Bifidobacterium*, *Streptococcus*, *Akkermansia*, and *Clostridium* genera were significantly decreased in COVID-19+ATB group (*p* < 0.05; logOR > −1) (Figure 5). The *Desulfovibrio* and *Bifidobacterium* genera were reduced in COVID-19+ATB, when compared with COVID-19 group (Figure 6). The *Bacteroides*, *Parabacteroides*, *Alistipes*, *Bilophila*, and *Barnesiella* genera were overrepresented in both groups (COVID-19 and COVID-19+ATB) (Figure S4). Figure S5 shows the overrepresented genera in COVID-19+ATB, and Figure S6 shows the underrepresented ones in COVID-19+ATB group.

## 4. Discussion

The intestinal microbiota are important for several physiological processes, including the modulation of both innate and adaptive immune responses that maintain a systemic immune homeostasis [35,36,37,38,39,40]. The role of intestinal dysbiosis in several inflammatory conditions have already been demonstrated, and for the past few decades an increasing body of evidence has pointed toward the microbiota as a major player in infectious diseases, including that of COVID-19 [41,42,43,44,45,46,47]. Here, we evaluated the intestinal microbiota in Brazilian patients in different post-COVID-19 periods and correlated it with clinical data and the antibiotic therapy used during the acute phase.

Although the first studies evaluating the intestinal microbiota in COVID-19 patients had a small number of samples, the available data suggest that dysbiosis and its influence on immune responses played an important role on the progression to a severe form of the disease and dysfunction of immune responses [23,24,25,26]. A shotgun sequencing study, carried out by Zuo et al. demonstrated significant changes in the microbiota of 15 hospitalized patients (1 mild, 9 moderate, 3 severe, 2 critical), with enrichment of opportunistic microorganisms in the antibiotic therapy group (N = 7 vs. 15 controls), including *Actinomyces viscosus*, *Bacteroides nordii*, and *Clostridium hathewayi*, and decrease of beneficial commensals, such as *Eubacterium rectale*, *Faecalibacterium prausnitzii*, *Dorea formicigenerans*, and *Ruminococcus obeum*. The intestinal dysbiosis persisted even after a negative SARS-CoV-2 test and respiratory symptoms resolution [23]. The relative abundance of *F. prausnitzii*, which favors an anti-inflammatory microenvironment [48], was inversely correlated with disease severity [23]. This was the first study showing dysbiosis in patients infected with SARS-CoV-2 during the acute phase. Another one, from the same group, identified *Collinsella aerofaciens*, *C. tanakaei*, *Morganella morganii*, and *Streptococcus infantis* in the microbiota from COVID-19 patients with SARS-CoV-2 fecal positivity (N = 7), after six days of viral clearance from respiratory samples. Patients with negative infectivity (N = 8) had increased SCFA-producing bacteria, including *Alistipes onderdonkii*, *Bacteroides stercoris*, and *Parabacteroides merdae* [24].

Similarly, a study conducted by Gu et al. evaluated the gut microbiota by 16S sequencing from 30 COVID-19 patients, 24 H1N1, and 30 controls. COVID-19 patients presented decreased microbiota richness and diversity, when compared with the control group, with a predominance of opportunistic genera, such as *Actinomyces*, *Erysipelatoclostridium*, *Rothia, Streptococcus*, and *Veillonella*, which correlated with CRP and D-dimer levels. In addition, COVID-19 samples had lower abundance of beneficial symbionts, *Agathobacter*, *Fusicatenibacter*, and *Roseburia*. This study excluded patients under antibiotic therapy and proposed 5 genera as a microbiota signature to differentiate COVID-19 from the control group (*Actinomyces*, *Erysipelatoclostridium*, *Fusicatenibacter*, *Intestinibacter*, *Romboutsia*) [25].

Concerning post-COVID-19, a shotgun sequencing analysis of the gut microbiota in COVID-19 (N = 100), control individuals (N = 78), and post-COVID-19 patients (N = 27), 30 days after viral clearance, showed dysbiosis in post-COVID-19 patients, independent of antibiotic therapy (14 received). There were no significant differences in Shannon index between acute COVID-19 and control groups, and researchers did not compare these previous groups with post COVID-19 samples. In addition, they detected decreased relative abundance of *Bifidobacterium*, *Eubacterium rectale*, and *F. prausnitzii* in stool samples from acute COVID-19, and these alterations persisted in post-COVID-19 patients. Finally, this study demonstrated correlations among dysbiosis, systemic inflammatory markers and disease severity, and was the first one to hypothesize that the persistence of intestinal dysbiosis in post-COVID-19 patients may be involved in the clinical manifestations months after the disease resolution [26].

In a study from Liu et al., intestinal dysbiosis persisted for six months after COVID-19 resolution. Researchers evaluated the gut microbiota by shotgun sequencing in post-COVID-19 patients (4 asymptomatic, 31 mild, 55 moderate, 10 severe) at one- (N = 64), six- (N = 68) and nine-month (N = 11) follow-up, and in 68 control individuals. Dysbiosis, with decreased microbiota diversity, in antibiotic-naïve COVID-19 patients were detected, when compared with the control group. Decreased Shannon index was observed in acute COVID-19, compared with control subjects and post-COVID-19 (one- and six-month follow-up). However, the Shannon index was increased in post-COVID-19, at six-month follow-up, when compared with the control group, which was similar to our study. The relative abundance of *Bifidobacterium* and *Ruminococcus* was significantly reduced in antibiotic-naïve patients. Differences between antibiotic-naïve and antibiotic-treated patients were not detected at the six-month follow-up [27]. Approximately 76% of patients in this study presented sequelae after acute COVID-19 (fatigue, memory and hair loss), and patients without eubiosis reestablishment were characterized by increased *Bacteroides vulgatus* and *Ruminococcus gnavus*, as well as decreased *F. prausnitzii* [27]. Similarly, Chen et al. investigated the gut microbiota by 16S sequencing in 30 COVID-19 patients at hospitalization and six months after discharge. Authors observed decreased microbiota richness in the acute disease compared with the control group (N = 30), and higher CRP and disease severity (in acute phase) in the same post-COVID-19 patients with lower microbiota diversity [49].

In a more recent study, using shotgun sequencing and mass spectrometry analysis, Zhang et al. evaluated the gut microbiota and metabolites in 66 hospitalized antibiotic-free COVID-19 patients (31 mild, 16 moderate, 19 severe/critical), 35 post-COVID-19 (30 days after discharge), and 70 controls. The microbiota composition in severe/critical COVID-19 patients was significantly different from that in the control group; *Bifidobacterium adolescentis*, *F. prausnitzii*, and *Ruminococcus bromii* were underrepresented, and pathways related to carbohydrate fermentation and SCFA production were impaired. The post-COVID-19 patients (15 mild, 17 moderate, 13 severe) also presented different microbiota function and composition, when compared with the control individuals. *B. adolescentis*, *F. prausnitzii*, and *R. bromii* were still reduced 30 days after discharge, as well as pathways associated with SCFA production and L-isoleucine biosynthesis. Interestingly, the lower abundance of *B. adolescentis* and *F. prausnitzii* in COVID-19 patients was associated with more severe symptoms [50].

In our study, we evaluated the intestinal microbiota in 149 post-COVID-19 patients (10 asymptomatic, 117 mild, 10 moderate, 12 severe), ranging from one- to eight- month follow-up, and 71 control samples. We detected significant differences in beta diversity in post-COVID-19 patients, when compared with the control group. To exclude the effect of antibiotics on the intestinal microbiota of patients who used them during the acute phase, we evaluated the groups separately (COVID-19 vs. COVID-19+ATB), and we identified differential relative abundance of some genera in the COVID-19 group, suggesting a microbiota signature related to the disease effects, including *Desulfovibrio*, *Haemophillus*, *Dialister*, and *Prevotella.* The *Desulfovibrio* genus comprises sulfate-reducing bacteria present in the human mouth and gut, and, due to hydrogen sulphide release, it has been associated with chronic periodontitis, inflammatory bowel diseases, and septic processes [51,52,53,54,55]. *Haemophillus* genus is predominantly found in the nasopharyngeal and lung microbiome and is associated with co-infections or secondary infections in COVID-19 [56]. Some *Haemophillus* species colonize the human intestines and are associated with conditions that include irritable bowel syndrome, multiple sclerosis, and neuropsychiatric disorders, through the gut–brain axis [57,58,59,60]. *Dialister* could be found in the human gut and the increased abundance was associated with neurological conditions, such as multiple sclerosis, depression, and attention-deficit and hyperactivity disorder [61,62,63]. Finally, *Prevotella* is one of the most abundant genera found in the human gut, and such increased abundance is associated with Th17-mediated mucosal inflammation, besides rheumatoid arthritis, metabolic disorders and low-grade systemic inflammation [64,65,66].

In addition to these data, some beneficial microbes, associated with gut health (*Bifidobacterium* and *Akkermansia*), were significantly decreased in patients that used antibiotics during the acute phase (COVID-19+ATB), thus suggesting that antibiotics are associated with long-term effects on the intestinal microbiota in post-COVID-19 patients. Antibiotics have significant impacts on the composition and diversity of the intestinal microbiota, with increased *Enterobacteriaceae* species, besides decreased *Bifidobacterium* and beneficial butyrate-producing species [67,68]. The use of antibiotics increased worldwide during the COVID-19 pandemic, particularly to treat severe cases and secondary lung infections [69], although patients also used it with the mild form of the disease, and even some asymptomatic patients, as described in our study. Antibiotics are not only involved in dysbiosis and antimicrobial resistance, but also in long-term consequences, such as gastrointestinal infections, weight gain, obesity, inflammatory bowel diseases, and colorectal cancer [68,69,70].

Therefore, we hypothesized that intestinal dysbiosis detected in post-COVID-19 patients, and the indiscriminate use of antibiotics during the acute phase, may be associated with post-COVID-19 syndromes. In our study, approximately 39% (N =58) of post-COVID-19 patients reported clinical manifestations after resolution of the acute phase, including fatigue, dyspnea, myalgia, alopecia, anxiety, memory loss, depression and persistent anosmia. The main strength of our study is the large sample size, which improves the statistical power to assess the differential relative abundances between the evaluated groups. However, our study had some limitations, such as a lack of data on dietary habits from the patients, and a lack of acute COVID-19 samples, which would allow us to conduct a temporal analysis of the microbiota. Moreover, there were some differences between this and other studies carried out with post-COVID-19 patients, such as study population (number of patients with different disease forms), control group, mean age, sex proportions, sequencing techniques, and collection time points of the post-COVID-19 samples, and they should be taken into account.

## 5. Conclusions

In this observational study, we detected some genera possibly associated with the post-COVID-19 dysbiosis, including *Desulfovibrio*, *Haemophillus*, *Dialister*, and *Prevotella*, in addition to decreased beneficial microbes associated with antibiotic-induced dysbiosis, including *Bifidobacterium* and *Akkermansia*. Such disease- and antibiotic-associated alterations may be related to the clinical manifestations of the post-COVID-19 (long COVID), and we suggest that the microbiota modulation may represent a target for recovery from the acute COVID-19 and a therapeutic approach for post-COVID-19 clinical manifestations.

## Figures and Tables

**Table 1:** Demographic, anthropometric, and clinical data from post-COVID-19 patients based on disease severity classification during the acute phase, and demographic and anthropometric data from control subjects.

Patients/ Controls	* Gender (F/M)	** Age (Mean ± SD)	*** BMI (kg/m^2^)	^§^ Total SARS-CoV-2 Antibodies	^−^ CRP (mg/dL)	^+^ Days Post-COVID-19 Mean
Asymptomatic N = 10 (6.7%)	8 F (80%) 2 M (20%)	36.5 ± 13.8	26.5 ± 4.7	29.2 ± 49.1	0.42 ± 0.27	72 (~10 weeks) (~2.4 months)
Mild N = 117 (78.5%)	78 F (66.7%) 39 M (33.3%)	40.4 ± 14.1	27.9 ± 5.0	68.6 ± 58.8 *p* = 0.047	0.56 ± 0.92	84 (~12 weeks) (~2.8 months)
Moderate N = 10 (6.7%)	7 F (70%) 3 M (30%)	49.6 ± 13.4	35.2 ± 5.3 *p* < 0.001	101.1 ± 57.5 *p* = 0.006	1.56 ± 1.68	81 (~11.5 weeks) (~2.7 months)
Severe N = 12 (8%)	5 F (41.7%) 7 M (58.3%)	43.7 ± 15.7	31.4 ± 4.7 *p* = 0.001	87.3 ± 43. 4 *p* = 0.032	3.22 ± 4.35	105 (~15 weeks) (~3.5 months)
Controls N = 71	51 F (71.8%) 20 M (28.2%)	46.1 ± 16.6	26 ± 4.7	ND	ND	NA

F: female; M: male; SD: standard deviation; BMI: body mass index; kg/m^2^: kilograms per square meters; CRP: C-reactive protein; mg/dL: milligrams per deciliter; ND: not determined. NA: Not applicable. * *p* = 0.110; ** *p* = 0.986; *** *p* < 0.05 (Controls vs. Moderate/Severe); **^§^** *p* = 0.006 (Asymptomatic vs. Mild/Moderate/Severe); **^−^** *p* = 0.282; **^+^** *p* = 0.505.

**Table 2:** Distribution of main symptoms and antibiotic therapy, based on disease severity classification, during the acute phase, besides sequelae in post-COVID-19 patients.

Patients	Diarrhea	Fever	Dyspnoea	Anosmia	Ageusia	Antibiotics	Sequelae
Asymptomatic (N = 10)	0	0	0	0	0	4 (40%)	0
Mild (N = 117)	37 (31.6%)	54 (46.1)	0	75 (64.1%)	59 (50.4%)	89 (76%)	41 (35%)
Moderate (N = 10)	4 (40%)	7 (70%)	10 (100%)	4 (40%)	6 (60%)	9 (90%)	6 (60%)
Severe (N = 12)	8 (66.7%)	10 (83.3%)	12 (100%)	5 (41.7%)	6 (50%)	12 (100%)	11 (91.6%)

## Data Availability

The sequences generated by 16S sequencing were deposited at the NCBI repository (BioProject ID: PRJNA758913) and can be accessed through https://www.ncbi.nlm.nih.gov/bioproject/758913 (accessed on 10 August 2020).

## Supplementary Materials

The following supporting information can be downloaded at: https://www.mdpi.com/article/10.3390/ijerph191610189/s1, Figure S1: Relative abundance of some overrepresented genera in post-COVID-19 patients; Figure S2: Relative abundance of some underrepresented genera in post-COVID-19 patients; Figure S3: Relative abundance of specific overrepresented genera in post-COVID-19 patients without antibiotic therapy; Figure S4: Relative abundance of some overrepresented genera in both post-COVID-19 and COVID-19+ATB group; Figure S5: Relative abundance of specific overrepresented genera in COVID-19+ATB group; Figure S6: Relative abundance of specific underrepresented genera in COVID-19+ATB group.
