# Supplementary figures and images for: Detection of Intestinal Dysbiosis in Post-COVID-19 Patients One to Eight Months after Acute Disease Resolution
Source: Int J Environ Res Public Health. 2022 Aug 17;19(16):10189. doi: 10.3390/ijerph191610189 (PMC9408204; doi:10.3390/ijerph191610189)

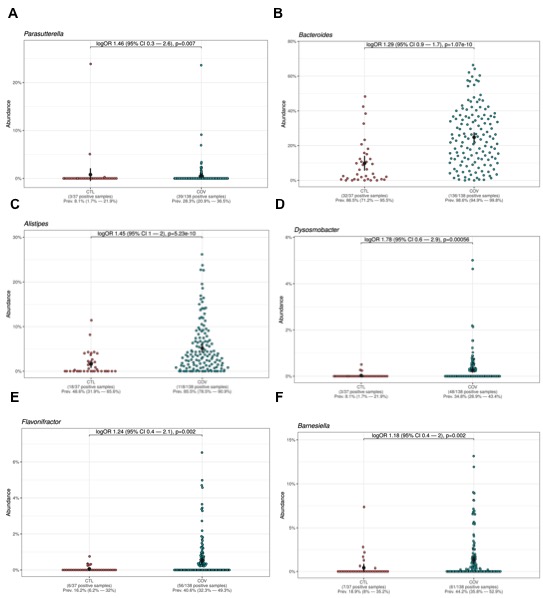

Supplement: Supplementary file 1 [file ijerph-19-10189-s001.zip › Figure S1--Relative abundance of some overrepresented genera in post-COVID-19 patients.tiff]

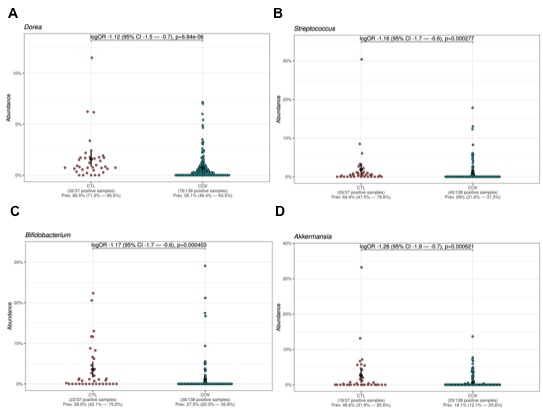

Supplement: Supplementary file 1 [file ijerph-19-10189-s001.zip › Figure S2--Relative abundance of some underrepresented genera in post-COVID-19 patients.tiff]

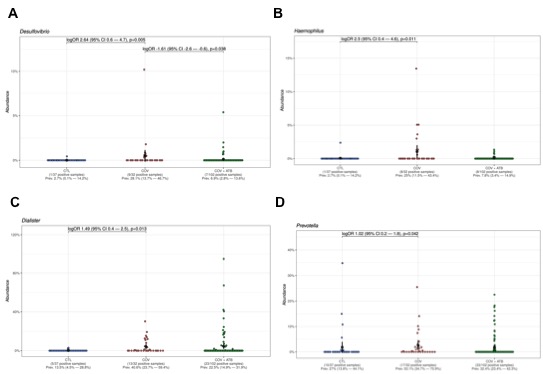

Supplement: Supplementary file 1 [file ijerph-19-10189-s001.zip › Figure S3--Relative abundance of specific overrepresented genera in post-COVID-19 patients without antibi-otic therapy.tiff]

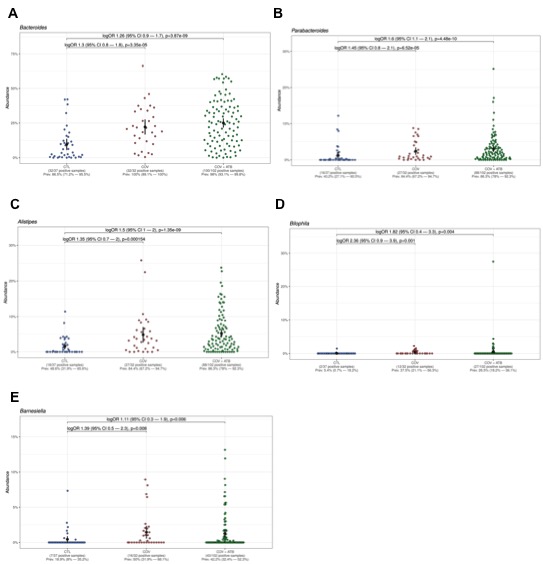

Supplement: Supplementary file 1 [file ijerph-19-10189-s001.zip › Figure S4--Relative abundance of some overrepresented genera in both post-COVID-19 and COVID-19+ATB group.tiff]

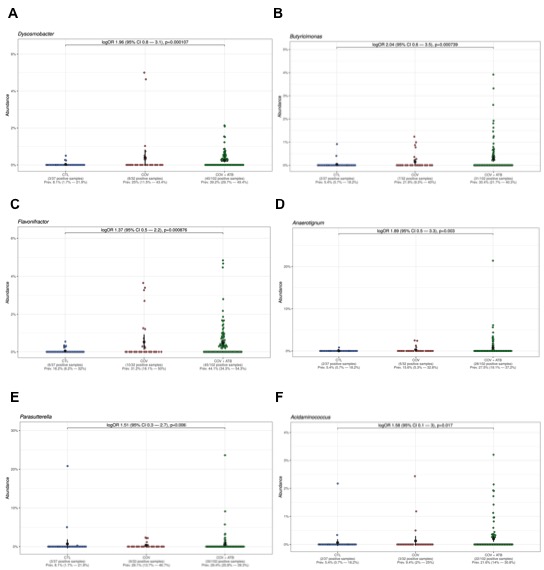

Supplement: Supplementary file 1 [file ijerph-19-10189-s001.zip › Figure S5--Relative abundance of specific overrepresented genera in COVID-19+ATB group.tiff]

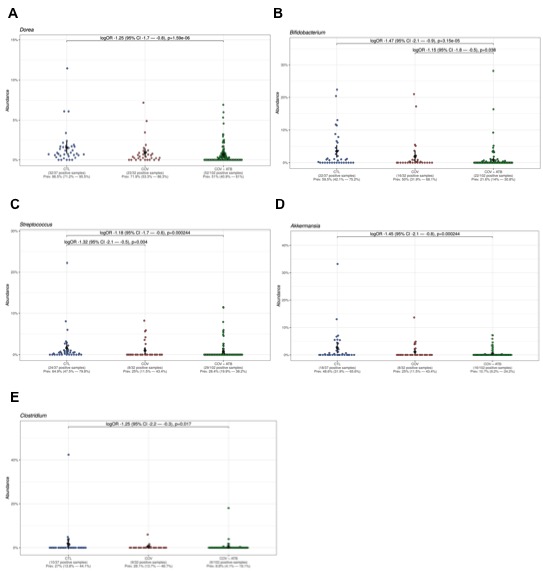

Supplement: Supplementary file 1 [file ijerph-19-10189-s001.zip › Figure S6--Relative abundance of specific underrepresented genera in COVID-19+ATB group.tiff]
